# Development of the Improving Process for the 3D Printed Structure

**DOI:** 10.1038/srep39852

**Published:** 2017-01-05

**Authors:** Kensuke Takagishi, Shinjiro Umezu

**Affiliations:** 1 Graduate School of Creative Science and Engineering, Waseda University, Department of Modern Mechanical Engineering, Shinjuku, Okubo 169-8555, Japan; 2 Undergraduate School of Creative Science and Engineering, Waseda University, Department of Modern Mechanical Engineering, Shinjuku, Okubo 169-8555, Japan

## Abstract

The authors focus on the Fused Deposition Modeling (FDM) 3D printer because the FDM 3D printer can print the utility resin material. It can print with low cost and therefore it is the most suitable for home 3D printer. The FDM 3D printer has the problem that it produces layer grooves on the surface of the 3D printed structure. Therefore the authors developed the 3D-Chemical Melting Finishing (3D-CMF) for removing layer grooves. In this method, a pen-style device is filled with a chemical able to dissolve the materials used for building 3D printed structures. By controlling the behavior of this pen-style device, the convex parts of layer grooves on the surface of the 3D printed structure are dissolved, which, in turn, fills the concave parts. In this study it proves the superiority of the 3D-CMF than conventional processing for the 3D printed structure. It proves utilizing the evaluation of the safety, selectively and stability. It confirms the improving of the 3D-CMF and it is confirmed utilizing the data of the surface roughness precision and the observation of the internal state and the evaluation of the mechanical characteristics.

We developed the 3D Chemically Melting Finishing (3D-CMF) method for the 3D printed structure for precision surface. 3D printing has been used as rapid prototyping tool in the industrial field. 3D printer was recently-popularized because the principle patent on the Fused Deposition Modeling (FDM)^1^,^2^,^3^,^4^,^5^,^6^,^7^,^8^ that was developed by Mr. S. Crump in 1989 was expired at 2007. As June of 2016, 3D printing technology is applied for following many fields. Artificial bone, artificial joint, and artificial organ in the bio medical technology field and solar cell in green technology field were fabricated by the 3D inkjet method^9^,^10^,^11^,^12^,^13^. Complex metal products were fabricated by the Selective Laser Sintering (SLS)^14^,^15^,^16^. Commercial plastic products were fabricated by the FDM. Among these 3D printing methods, the FDM will be explosively sold by the following reasons. Acrylonitrile-butadiene-styrene (ABS)^17^,^18^,^19^,^20^,^21^,^22^, Poly-Lactic Acid (PLA), and other polymers are used for the filament of the FDM. The cost of the 3D printing machine is cheap (several hundred US$ at June of 2016). High skill to print is not required and the 3D structure is fabricated with PC and the Standard Triangulated Language (STL)^23^ data. In spite of the former merits, the market of the FDM was now gradually increased. When the layer was printed on the printed layer utilizing the FDM, layer grooves was formed. The layer grooves generated following two problems. First one was poor appearance due to the surface cracks. Second one was low rigidity due to the inner pores. So, the FDM was limited for fabricating the 3D sample. Because the main applications of the FDM were anime figurines and model structures, the customer of the FDM was not the public, but someone whose hobby was fabricating 3D anime figures, architect who used the building model, and engineers who used the prototype for discussion.

It was essential to create new and cheap method to finish the surface of the 3D printed structure to promote the 3D printer for home use. With the method, everyone can precisely print their original products those shape depends on their own design (for example, earphone, glasses, grip, helmet those match the shape of the individual). When the FDM was applied for the fabrication of the products, low cost 3D structure was printed with rough surface due to the layer grooves that was generated by the former two reasons. However, the rough surface should be improved for fabricating the products for daily use. So, the method to remove the surface layer grooves, make the smooth surface, and improve the strength is required. In general, the hybrid process of surface finishing and coating was applied. When the surface finishing was applied for the 3D printed structure, there are following two problems. The first one was impossible to remove the inner layer grooves. The stiffness was low because of the inner layer grooves. The second one was difficulty to apply uniform treatment on the complex surface because the polishing was easy to remove the peak of the surface and difficult to fill the bottom of the surface. So, usually coating process was applied to fill the bottom after the polishing process. However, easiness of the 3D printer was drastically reduced because the two additional processes were applied, and the cost and the time were increased. The second one was possibility to peel off the coating. Peeling off sometimes happened. because the concentrated stress was applied at the boundary surface between the 3D structure and coating due to different mechanical and chemical properties. The peeling off was critically negative for appearance.

There have been a variety of researches as the way to resolve the layer grooves that occur in this 3D printed structure.

3D printer was initiated by the Stereo Lithography Apparatus (SLA)^24^,^25^,^26^,^27^,^28^,^29^,^30^,^31^ method that was invented by Mr. Kodama at 1981, by Dr. A. Herbert at 1982. Charles W. Hull got the license at 1986, established 3D systems co., and succeeded in commercialization. The fabrication process of the SLA was shown as follows. In the case that the container was filled with the UV-curable resin, the resin was solidification by the radiated UV light. When the light was moved in xyz directions, 3D structure was fabricated. The effort involved during fabrication was similar to that in FDM, with the price of the device also similar since the expiry of the patent for the core technology in 2014. These facts make it suitable for home use. This method has been improved so that the stacking pitch of 3D printed structures can be fabricated at dozens of μm. Therefore, it superficially prevents the occurrence of layer grooves on 3D printed structures; however, surface cracks cannot be removed because of welding and ejection failures. Furthermore, with this method, the fabricated materials are limited to photo-curing resins that are significantly inferior to ABS resins (although they can be used for FDM) with respect to price, strength and quality management owing to the nature of the fabrication method. Therefore, the utilization of 3D printed structures prepared with SLA on a daily basis is quite difficult, and there are issues in using home 3D printers from the perspective of material utility.

Dieste *et al*. from Fundación Aitiip developed a method that automatically polished complex shapes^32^,^33^,^34^,^35^. With this method, the polishing process can be efficiently performed for complex 3D printed structures. This method is also capable of removing majority of layer grooves on the surface of 3D printed structures. Some 3D printers are actually equipped with a polishing device; however, the devices required for this method are expensive, thereby offsetting the advantages of FDM 3D printers (i.e. low cost and simplicity). According to its working principle, the polishing of 3D printed structures creates dust, which is not adequate for households and must, therefore, be recovered. However, adding a device to recover dust further increases costs and effort. These issues are important in distributing home 3D printers in general households.

He *et al*. of Zhejiang University proposed a method wherein the surface was melted with acetone and a solvent was used for removing the layer grooves from the ABS resin used for 3D printed structures^36^,^37^,^38^. The solvent was vaporized in the device, and a FDM 3D printed structure was placed inside. The vaporized solvent in the device uniformly melted the surface of the 3D printed structure, making the layer grooves less noticeable. As the surface melts, the material, previously converted into a liquid gel, penetrated the surface cracks. Thus, this method is considered to have a certain level of effectiveness in removing surface cracks as well; however, this method has two issues. First, it does not allow selective treatment. Thus, the vaporized solvent treats objects uniformly, thereby potentially reaching undesired areas. The second issue is similar to that found in painting, i.e., the fabrication of the device requires effort. As large volumes of inflammable acetone are used, there are significant safety risks associated with the use of this home 3D printer device.

Considering the utility of the materials and the costs associated with the preparation, FDM is the most suitable method for a home 3D printer. The biggest issue with FDM 3D printed structures is the layer grooves that occur in 3D modelling while the lamination of the filament melts upon heating. (there are FDM 3D printer that is high precision and does not cause layer grooves but as they are expensive and take long time to manufacture and are not suitable for home usage, we are focusing on cheap FDM 3D printer.) After printing with this type FDM 3D printer, the surfaces are normally smoothed by polishing; however, the layer grooves in the 3D printed structures have concave–convex shapes, and polishing cannot reach these concave areas. Therefore, these areas are filled by painting. By applying this two-step process, the layer grooves are removed and a 3D printed structure with a clean surface can be prepared, however, the long time for processing and the high cost makes it unsuitable as a final product for home 3D printers. Thus, we assumed that these issues could be solved if the layer grooves could be removed with a single, lost-cost process. Focusing on the CMF process, we proposed a 3D-CMF method that removes the layer grooves in 3D printed structures with a single low-cost process.

A principle diagram of this 3D-CMF method is shown in Fig. 1. In this method, a pen-style device is filled with a chemical able to dissolve the materials used for building the 3D printed structures. By controlling the behaviour of this pen-style device, the convex parts of the layer grooves on the surface of the 3D printed structure are dissolved, which, in turn, fills the concave parts. The layer grooves are thus smoothed. Using this pen-style device, a localized removal of the layer grooves in 3D printed structures is possible, and the amount of solvent used can be kept to a safe level. Unlike polishing, as this method does not need to shave the materials, it does not create dust. In this study, a pen-style device was filled with acetone (an organic solvent that can dissolve the ABS resin commonly used as a filament for FDM 3D printers), thereby creating a 3D-CMF treatment mechanism. We report our findings on the basic characteristics of 3D-CMF, the improvement in 3D printed structures with 3D-CMF and its superior characteristics compared with other treatments.

## Results

### The fundamental characteristics of the 3D-CMF

One of the factors hindering the widespread use of 3D printers is the outer appearance of the 3D printed structures which is rough because of the layer grooves. At present, the outer appearance of the 3D printed structures cannot be improved without sacrificing the low cost and ease of the 3D printer. Thus, we proposed a method to improve the outer appearance by enhancing the precision of the surface roughness of the 3D printed structures upon removal of the layer grooves with a pen-type 3D-CMF device. Using this method, the surface of the 3D printed structures can be improved at a low cost.

**Figure 2:**
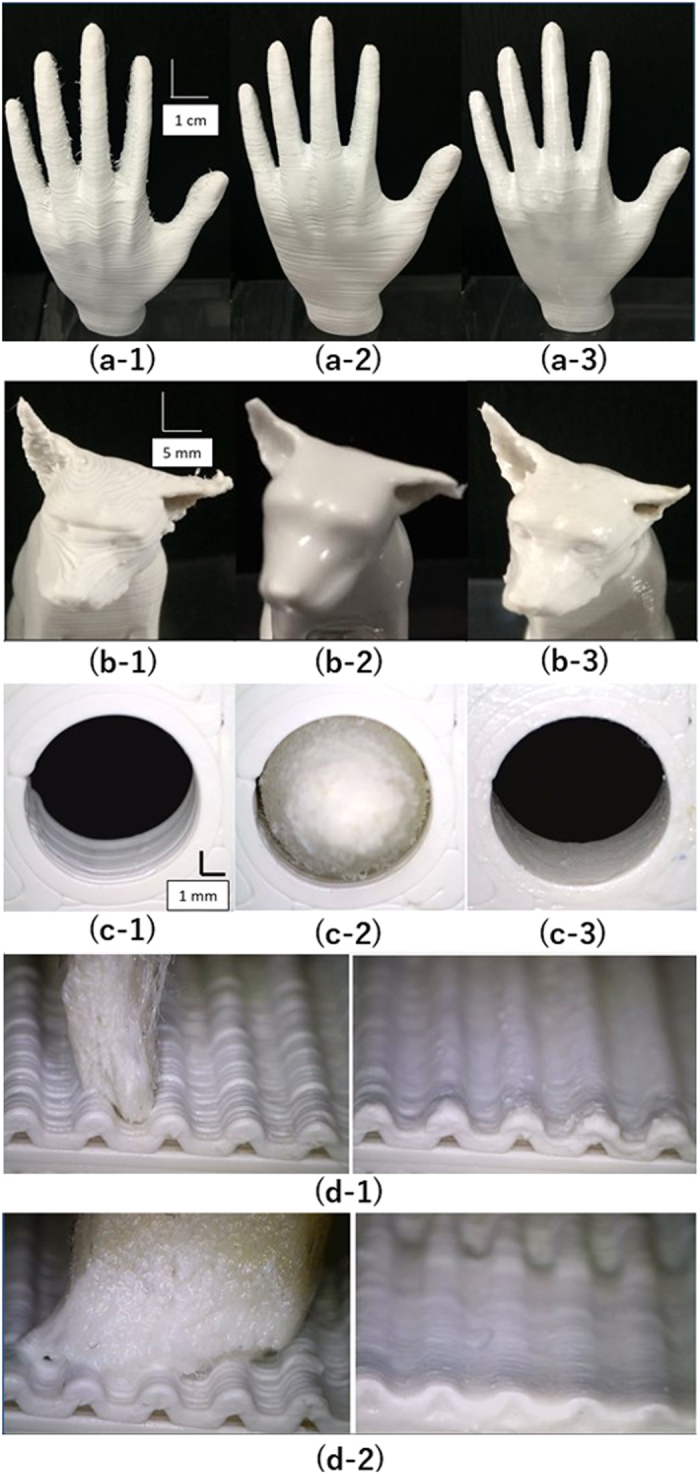
The 3D-CMF processing for the various complex 3D printed structures.

The surface of the 3D printed structures was improved with 3D-CMF. Figure 2(a) shows samples of a 3D printed structure simulating a human left hand. The sample undergoing conventional polishing and painting for layer grooves on 3D printed structure showed luster in some areas, probably because painting was performed only on those areas that were polished. Although the concave areas could not be accessed easily, polishing on the surface of the 3D printed structure with free surface was effective on protrusions. The treatment is thus not consistent, and only some parts of the sample were treated. In contrast, the sample treated with 3D-CMF showed luster on the whole surface. As felt was used on the tip of the pen during the 3D-CMF treatment mechanism, it was able to respond to the shape with free surface, thereby providing consistency to the treatment. Furthermore, the 3D-CMF does not create dust (potentially harmful to humans) while avoiding any negative impact on the surface condition. Thus, performing 3D-CMF on the 3D printed structures with free surface uniformly improved the surface roughness precision. In addition, it was superior compared to conventional polishing and painting methods.

In principle, 3D-CMF allows for a selective treatment of the 3D printed structures. We verified the superior characteristics of this method by selectively performing 3D-CMF on 3D printed structures with a complex shape and compared it with other treatments. Figure 2(b) shows 3D printed structure samples simulating a dog. The two samples undergoing surface treatment showed improvements compared to the untreated sample; however, one can realize that the eye lines vanish when focusing around the eyes of the dog that was treated with acetone vapor. In contrast, 3D-CMF improved the surface roughness while maintaining the eyes by performing a selective treatment. Therefore, it was verified that 3D-CMF can selectively treat complex-shaped 3D printed structures. Figure 2(c) shows how 3D-CMF is conducted to hole shape. It can be confirmed that the layer grooves inside the hole is being removed by adapting the shape of the pen tip to the hole. Figure 2(d) shows that by changing the pen tip, it is able to correspond to the wave structure shape. (d-1) shows how the pen tip is not suited for the structure and cannot correspond with the structure. (d-2) shows that by changing the pen tip, it has corresponded with the structure. The pressing force of the 3D-CMF performed on the complex 3D printed structure, shown in Fig. 2, needs to be set at a proper range in order to enable all area 3D-CMF. The force of 3D-CMF was applied in a range between the determined maximum and minimum force. (see Supplementary Fig. S1).

**Figure 3:**
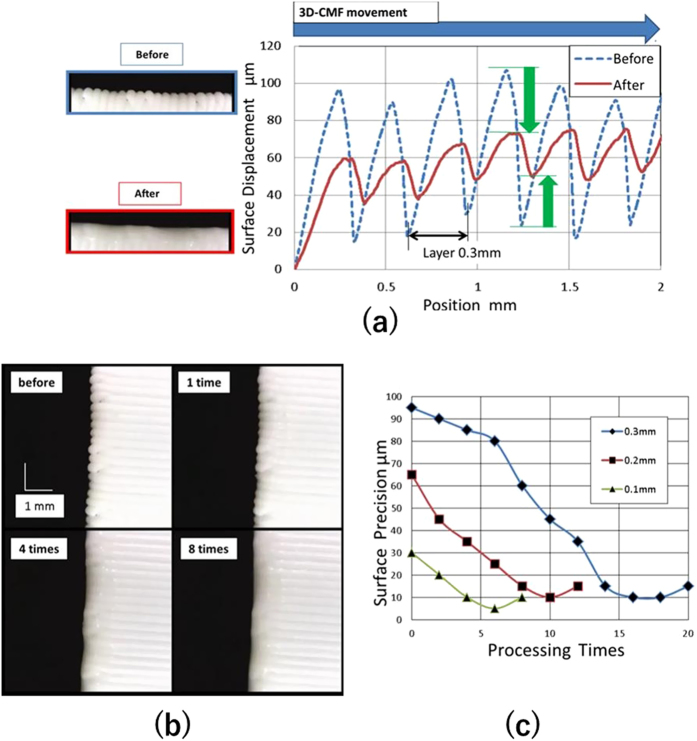
The fundamental characteristics of the 3D-CMF.

We were able to visually confirm that the layer grooves on the surface of 3D printed structures can be removed using 3D-CMF. Next, we numerically verified the basic characteristics of the melted protrusions, filling concave areas and, in turn, improving the surface roughness precision by smoothing. Figure 3(a) shows the surface displacement of a test piece before and after 3D-CMF measured with a laser displacement sensor. The surface displacement prior to the treatment confirmed that the interval between the highest and lowest points of the layer grooves was equal to the lamination pitch of 0.3 [mm]. As we limited the movement of the pen-style treatment mechanism to only one direction during 3D-CMF, the highest and lowest points of the layer grooves shifted to the direction of the movement of the pen-style mechanism. The height of the protruding layer groove decreased, while that of the base of the grooves increased. Overall, the Rz (ten-point average roughness) of the test piece surface improved from approximately 85 to 20 [μm] after the treatment. Therefore, we verified that the basic characteristics of this method (i.e. dissolving protrusions and filling the concave parts with melted materials) were correctly assumed.

In addition, based on the principle of 3D-CMF, the layer grooves on the surface became smoother as a result of the treatment. This was confirmed by a capture of a 3D-CMF treatment with a microscope (Fig. 3(b)) in which the layer grooves on the surface of a 3D printed structure were progressively removed with the treatment rounds. The lamination pitch of the test piece was 0.3 [mm]. The top-right image, taken after one round of treatment, shows the solvent painted on the surface of the test piece. The lower-left image shows a smoothed surface after four treatment rounds. The lower-right image, taken after eight treatment rounds, reveals that the layer grooves were removed as a result of the treatment. The layer grooves were confirmed to be progressively removed after each round during 3D-CMF.

Similarly, we confirmed that the surface displacement changed after each treatment round using a laser displacement sensor. The three types of test pieces (lamination pitches of 0.3, 0.2 and 0.1 [mm]) were treated once. At this point, the surface displacement was measured using a laser displacement sensor, and the results are shown in Fig. 3(c). The Rz decreased for all the lamination pitches undergoing more treatment rounds. When the lamination pitch was 0.3 [mm], the initial and final (nine rounds) Rz values were 95 and 10 [μm], respectively. When the lamination pitch was 0.2 [mm], the initial and final (six rounds) Rz values were 65 and 10 [μm], respectively. When the lamination pitch was 0.1 [mm], the initial and final (five rounds) Rz values were 30 and 5 [μm], respectively. Lower the lamination pitch, lower was the number of treatments required to smooth the surface. This can be explained in terms of the size of the layer grooves which depends on the lamination pitch. In addition, Rz started to increase after recording the minimum value since the material melted once 3D-CMF completely filled the layer grooves, and further treatment on the surface of a 3D printed structure made the surface rough again.

### The Reforming of the surface material according to the 3D-CMF

**Figure 4:**
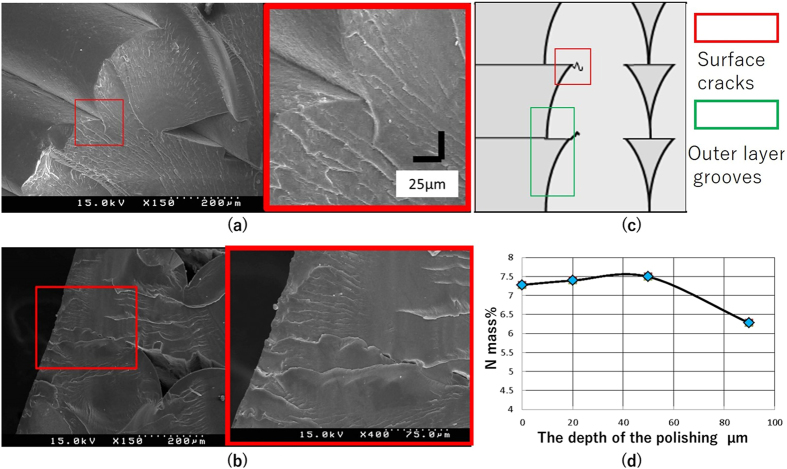
The change in the internal state of the 3D printed structure by the 3D-CMF.

The surface layer of the 3D printed structures was modified by applying 3D-CMF. This change was examined by Field Emission Scanning Electron Microscope (FE-SEM) and X-ray Fluorescence (XRF). XRF is a device to investigate the element content of the material surface. Figure 4(a) shows the image of the layer grooves on the surface layer by observing the cross section of a 3D printed structure. (Figure 4(a) shows the image of the outer layer grooves and surface cracks on the surface layer observed by FE-SEM). The left image shows the cross section prior to 3D-CMF. The layer grooves can be seen on the left side. The surface cracks, which could not be observed from the surface of a 3D printed structure, were seen in the central area. The right image shows an enlarged view of the surface cracks. These surface cracks became a unique breaking factor for the 3D printed structures, thereby decreasing their strength.

Figure 4(b) shows the cross section of a test piece after 3D-CMF observed by FE-SEM. The left image shows the cross section of a 3D printed structure after 3D-CMF. The layer grooves smoothed by 3D-CMF were visible on the left side. The layer grooves inside the red frame in the central part were filled. The right figure shows an enlarged view of the filled surface cracks showing surface cracks filled with melted materials. It also shows that the melted ABS resin layer is approximately 100 [μm] thick. This melted ABS resin filled the surface cracks that cause breaks in the 3D printed structures, decreasing the likelihood of breaking and increasing the strength of these structures.

Figure 4(c) shows what is outer layer grooves and surface cracks by using schematic diagram. Outer layer grooves are the wavelike grooves that appear on the surface of the material caused by lamination and surface cracks generate further inside outer layer grooves. We have been confirming visually surface cracks being removed by 3D-CMF process. Herein, a layer of melted material on the surface of the 3D printed structures was compositionally determined through 3D-CMF. The acetone melting process used herein for the ABS resin is valid for all the deposits except for butadiene rubber deposits without melting. Considering the surface layer (approximately 100[μm]) melted upon 3D-CMF, a layer with a high concentration of butadiene rubber and a low concentration of acrylonitrile and styrene would be formed in the deeper area, while a layer with a low concentration of butadiene rubber and high concentrations of acrylonitrile and styrene would be formed in the shallower area. Only acrylonitrile, which is one of the three organic matters constituting the ABS resin, contains nitrogen (N) as a constituent element. Therefore, as a way to compositionally confirm the presence of a layer of melted materials on the surface, we measured the mass content (%) of N using a X-ray fluorescence analysis (XRF) and roughly determined the thickness of the melted material layer. Then, the test piece was polished after 3D-CMF treatment at depths of 0, 20, 50, and 90 [μm], and each piece was measured by XRF. Figure 4(c) shows the relationship between the mass % of N as measured by XRF and the depth of polishing. The N content remained nearly unchanged at polishing depths of 0, 20 and 50 [μm]; however, at polishing depths of 90 [μm], the N content decreased significantly. This indicates that the layer containing a high butadiene concentration starts at a depth of 90 [μm]. In other words, the melted ABS resin layer is verified to be located at a depth of 90 [μm].

### The Strength of the 3D printed structure that were processed 3D-CMF

**Figure 5:**
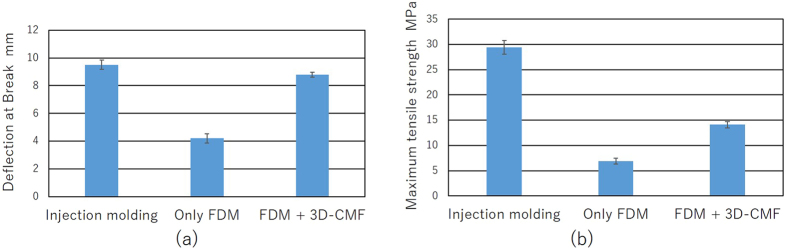
The evaluation of the improving in strength of the 3D printed structure by the 3D-CMF.

We confirmed that 3D-CMF achieves filling of the layer grooves on the surface layer of 3D printed structures, creating a layer of melted materials on the surface. As surface cracks are filled and a layer with less breaking factor forms on the surface, the 3D printed structures can withstand further deformation. Therefore, we prepared a 3D printer provided with a cantilever and performed a bending test. The amount of deflection at the break was measured, and the results are shown in Fig. 5(a). The amount of deflection of the untreated cantilever after fabrication with a 3D printer was 4.21 [mm]. The amount of deflection of the cantilever after 3D-CMF treatment was 8.78 [mm]. These results show that a 3D-CMF treatment on the 3D printed structures reduces the breaking factor and changes the structures, making them more resistant to deformation. Furthermore, we calculated the amount of deflection for a cantilever prepared by injection moulding using an analysis of the stress concentration zone in the cantilever. The result was 9.51 [mm] at the break. The purpose of the bending test was to measure deformation of the structure. The results of the bending test are caused by either or both of the improvement of the maximum tensile stress and maximum compressive stress. Therefore, we carried out a tensile test to measure the change in the maximum tensile stress. The result is shown in Fig. 5(b). The amount of maximum tensile strength of the untreated test piece after fabrication with a 3D printer was 6.89 [MPa]. The amount of maximum tensile strength of the test pieces after 3D-CMF treatment was 14.01 [MPa]. And we calculated the amount of maximum tensile strength for a test pieces prepared by injection moulding using an analysis of the stress concentration zone in the cantilever. The result was 29.40 [MPa]. It could be seen that maximum tensile stress is improved by 3D-CMF from the results of the tensile test. This result was caused because the surface cracks were filled. Also, the 3D printed structures processed 3D-CMF could acquire the strength that is inferior to the injection moulding that is much better than only 3D printed. This is because the printed layer is easy to be peeled. The layer grooves in 3D printed structures showed a significant impact on the strength of the 3D printed structures. 3D-CMF can remove such grooves, thereby improving the surface roughness precision while increasing the strength of the 3D printed structures. 3D-CMF treated 3D printed structures can handle even larger deformations.

## Discussion

**Figure 6:**
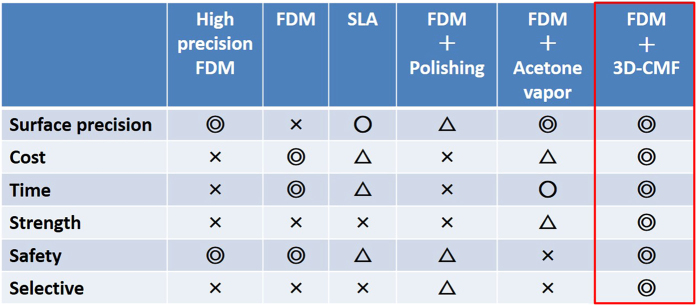
The comparison the 3D-CMF and previous processing in the 6 aspects.

We verified that 3D-CMF provides a consistent treatment for 3D printed structures with a free-form surface without producing harmful dust during the process. We showed this method to have superior characteristics as compared to the conventional polishing and painting treatments. In addition, we verified that 3D-CMF has superior performance for the selective treatment of 3D printed structures with complex shapes compared with the acetone vapour treatment, thereby exhibiting its diverse utility. By confirming the improvement in surface roughness precision of the 3D printed structure (both visually and numerically) using the Rz, we numerically verified that 3D-CMF drastically improves the surface condition of the 3D printed structures. By observing internal changes in the 3D printed structures made by 3D-CMF, we confirmed that the effects of 3D-CMF are not limited to improving the surface roughness precision. Thus, by analysing the materials of the surface layer changed by 3D-CMF, we measured the depth of the 3D-CMF effect on the 3D printed structures. We compositionally verified that the quality of the 3D printed structures was changed. The examination of the mechanical characteristics of the 3D printed structures after 3D-CMF treatment confirmed that this treatment produced 3D printed structures which more resistant to deformation. Thus, we showed that 3D-CMF safely removes layer grooves from the 3D printed structures at a low cost while increasing their strength. Therefore, the 3D-CMF treatment is an ideal choice in a home 3D printing environment, where cheap and safe fabrication of 3D printed structures resistant to deformation yet consistent with users’ image is desired. Figure 6 summarizes the above results.

## Methods

### The fundamental characteristics of the 3D-CMF

**Figure 7:**
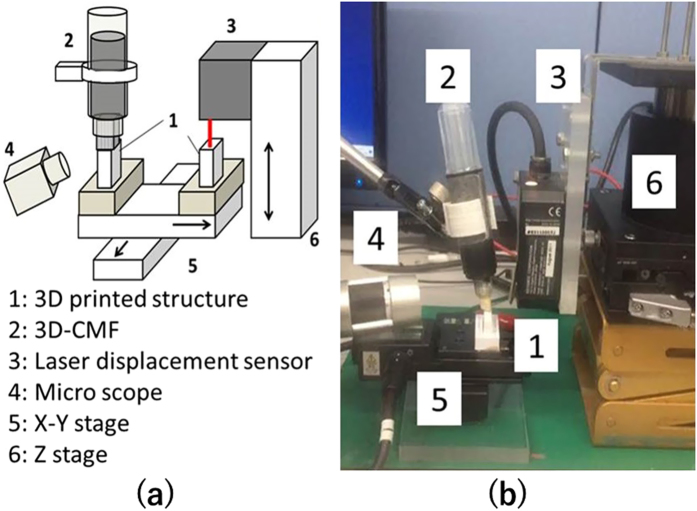
The device that verify the change surface state of the 3D printed structure by the 3D-CMF.

It aims to grasp the fundamental characteristics of the 3D-CMF. The experiment apparatus that we developed is shown in Fig. 7. Samples were printed ABS resin as a material. The STL data of the sample shaped 5*15*25 are modeled utilizing 3D-CAD (Dassault Systems, France, Solidworks 2011). Samples are printed utilizing the FDM 3D printer (abee, Tokyo, SCOOVO X9). The head speed of the 3D printer was set to 30 [mm/s]. Acetone dissolves the ABS resin. Therefore we use acetone for the solvent. As ABS resin solute to form a homogeneous and dispersed in acetone as a solvent. ABS resin is a polymer of acrylonitrile, butadiene and styrene. Only acrylonitrile and styrene is dissolved in acetone. Therefore the viscosity in a state of dissolving the ABS resin in acetone becomes higher than the conventional liquid. This high viscosity makes it easy to control in a dissolved state. Utilizing the liquid ink type marker pen (KOKUYO, Tokyo, Yokumie-ru) for the pen type processing mechanism. Acetone put into the pen as solvent ink. It adjust the amount of acetone that exits at the pen tip by the increasing the density of the sponge in the pen. The surface displacement is measured utilizing laser displacement sensor (KEYENCE, Osaka, LK-H008). The shooting of the changes in the surface is utilizing the Micro scope (Sanko, Tokyo, Dino capture Premium Lite). The processing, the measuring and the shooting are carried out in the same system. Therefore the deviation of the measuring is minimized.

### The Reforming of the surface material according to the 3D-CMF

The shooting of the changes of the internal state that is produced by 3D-CMF is utilizing FE-SEM (HITACHI, Ibaraki, S-4500S). It is necessary to expose the cross section of the sample when shooting with FE-SEM. It is necessary to expose the sample cross section not to cause plastic deformation. Therefore, the test piece is broken under the glass transition point of ABS resin (−80~90 [°C]). This temperature environment is created by liquid nitrogen. The identification of the ABS resin layer that is produced by 3D-CMF is utilizing XRF (RIGAKU, Tokyo, ZSX Primus II). Also this time, the error becomes smaller if the measurement range becomes larger. Therefore, the measurement range was set at a maximum of φ30 [mm].

### The Strength of the 3D printed structure that were processed 3D-CMF

**Figure 8:**
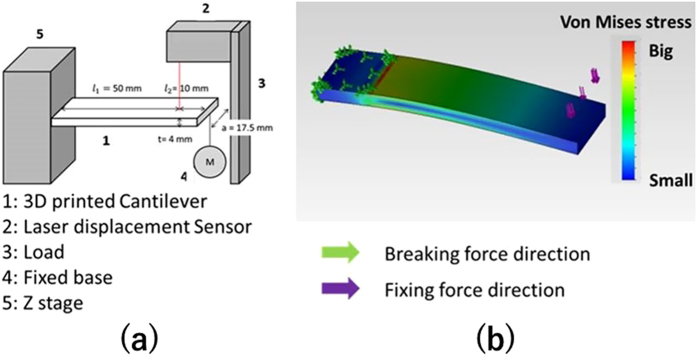
The device of the bending test mechanism of cantilever.

It aimed to grasp the fundamental characteristics of the 3D-CMF. The experiment apparatus that we developed is shown in Fig. 8(a). Cantilevers were printed ABS resin as a material. The STL data of the sample that shape is 75*17.5*4 [mm] is modeled utilizing 3D-CAD (Dassault Systems, France, Solidworks 2011). Cantilevers are printed utilizing the FDM 3D printer (abee, Tokyo, SCOOVO X9). The head speed of the 3D printer was set to 30 [mm/s]. The measurement of the deflection at break is measured utilizing laser displacement sensor (KEYENCE, Osaka, LB-040). The cantilever is fixed as shown in Fig. 8(a). Fix the laser displacement sensor and the measurement position set as Fig. 8(a). Attach the sling to hang the load at the free end of the cantilever. Attach the load which can break the cantilever reliably at the end of the sling. Prevent shaking pressed by hand. Start the measurement utilizing the laser displacement sensor. And it moves the load down slowly. The cantilever is broken when it could not bear the load. Record the amount of deflection at break. It measures the amount of deflection at break of the 3D printed cantilevers in the above methods. Analyze data of the cantilever that is produced with injection molding. The 3D-CAD (Dassault Systems, France, Solidworks 2011) is used for the analysis. First, identify the stress concentration portion. Next calculate the load amount (M) that reaches the maximum bending stress at the stress concentration portion. And then it calculates amount of the deflection when add the load (M). The stress concentration zone is the fixed end of the cantilever. It is shown in Fig. 8(b). Young’s modulus of the ABS resin (E) is 2.65 [GPa]. The maximum bending stress of the ABS resin (F) is 68.5 [MPa]. Section modulus (Z) and moment of inertia is calculated from the shape of the cantilever.


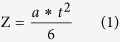



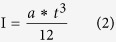


The load when it reaching the maximum bending stress (F) at the stress concentration zone (M) is calculated utilizing the following formula and (Z) and (I).


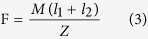



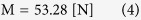


Calculate the amount of deflection when adds the breaking load.


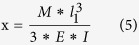



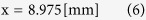


This value is used as a bending test result of the cantilever that was created in injection molding.

## Additional Information

**How to cite this article:** Takagishi, K. and Umezu, S. Development of the Improving Process for the 3D Printed Structure. *Sci. Rep.* **7**, 39852; doi: 10.1038/srep39852 (2017).

**Publisher's note:** Springer Nature remains neutral with regard to jurisdictional claims in published maps and institutional affiliations.

## Supplementary Material

Supplementary Figure 1

Supplementary Information

## Figures and Tables

**Figure 1:**
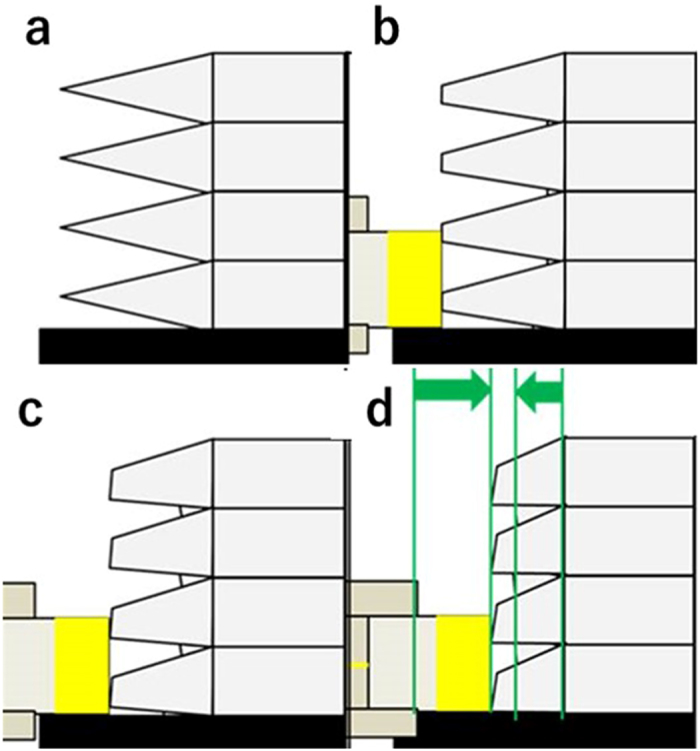
The principle diagram of the 3D-CMF.
