# Supplementary figures and images for: Development of the Improving Process for the 3D Printed Structure
Source: Sci Rep. 2017 Jan 5;7:39852. doi: 10.1038/srep39852 (PMC5215526; doi:10.1038/srep39852)

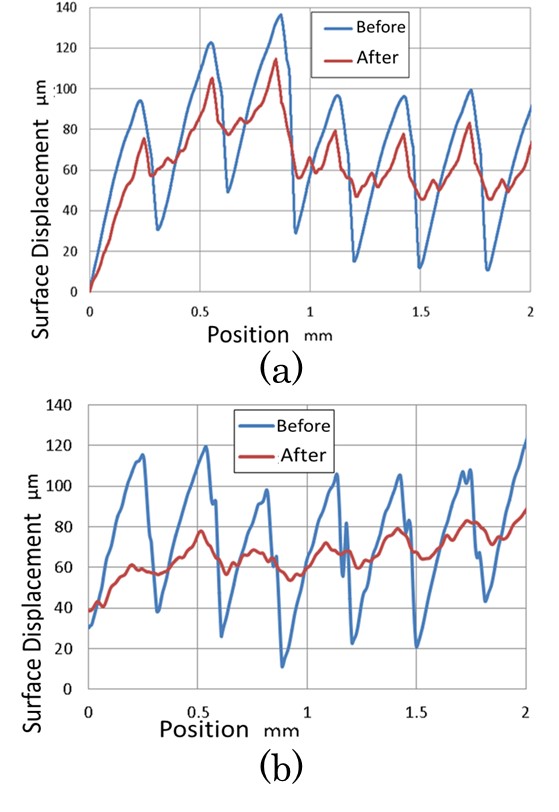

Supplement: Supplementary Figure 1 [file srep39852-s1.jpg]
