# Supplementary material for: Development of the Improving Process for the 3D Printed Structure
Source: Sci Rep. 2017 Jan 5;7:39852. doi: 10.1038/srep39852 (PMC5215526; doi:10.1038/srep39852)
Supplement: Supplementary Information [file srep39852-s2.pdf]

Development of the Improving Process for the 3D Printed Structure

Kensuke Takagishi[1*], **Shinjiro Umezu**[2**]

[1]Graduate School of Creative Science and Engineering, Waseda University, Department of Modern Mechanical Engineering, Shinjuku, Okubo169-8555, Japan
[2]Undergraduate School of Creative Science and Engineering, Waseda University, Department of Modern Mechanical Engineering, Shinjuku, Okubo169-8555, Japan
[*]ken1017suke@ruri.waseda.jp
[**]umeshin@waseda.jp
these authors contributed equally to this work

Supplementary Information

We have controlled 3D-CMF pen manually when applying process to a complicated shape such as hands and dogs in Figure 2.

We have confirmed the process to be effective in the area of manual process beforehand. Manually 3D-CMF processed in a state that the pen shape does not change. The tip shape changes when the pressing force is larger than 1.568N. It processed a lateral aspect of 3D printed cubic automatically controlling the pressing force at 0.1N and 1.568N. And it processed 8 times in both directions.

The results are shown in Supplementary Figure S1. This result shows that as the pressing force stays in the area between 0.1N and 1.568N, the process is effective.

Although it depends on the strength of the pressing force, we have confirmed that up until a certain amount of times processed, surface roughness is improved by the process, but after that certain times, surface roughness does not change.

Manually 3D-CMF processed the hand and dog structure 10 times keeping pressing force under 1.568N. However in minute structure, 3D-CMF process diminishes the minute structure if the process times are more than needed to improving surface roughness. Therefore minute structure requires close monitoring when processing. We have processed minute structure such as dog eye and base of finger gently 15 times while watching the surface micro structures.

Supplementary Figure List

Supplementary Figure S1. Surface displacement by automatic control of 3D-CMF ((a):Pressing force was 0.1 N (b)Pressing force was 1.568 N)
